# Design and Optimization of High-Responsivity High-Speed Ge/Si Avalanche Photodiode in the C+L Band

**DOI:** 10.3390/mi14010108

**Published:** 2022-12-30

**Authors:** Chuan Li, Xinyu Li, Yan Cai, Wei Wang, Mingbin Yu

**Affiliations:** 1 State Key Laboratory of Functional Materials for Informatics, Shanghai Institute of Microsystem and Information Technology, Chinese Academy of Sciences, Shanghai 200050, China; 2 University of Chinese Academy of Sciences, Beijing 100049, China; 3 Shanghai Industrial Technology Research Institute, Shanghai 201800, China

**Keywords:** silicon photonics, avalanche photodiodes, mechanical strain, Ge/Si

## Abstract

We present the design of Ge/Si avalanche photodetectors with SiN stressor-induced Ge strain for the C+L band light detection. By optimizing the placement position and thickness of the SiN layer with compressive stress, a uniform strain distribution with a maximum magnitude of 0.59% was achieved in Ge. The surface-illuminated APDs have been studied in respect of the photo-dark current, responsivity, gain, and 3-dB bandwidth. After introducing SiN stressor, the APD exhibits high primary responsivity of 0.80 A/W at 1.55 μm, 0.72 A/W at 1.625 μm, and 3-dB bandwidth of 17.5 GHz. The increased tensile strain in Ge can significantly improve the responsivity and broaden the response band of the device. This work provides a constructive approach to realizing high-responsivity high-speed Ge/Si APD working in the C+L band.

## 1. Introduction

In view of the high optical absorption of germanium in the near-infrared band, the research on germanium/silicon avalanche photodiodes (APDs) mostly focuses on the wavelength of 1.31 μm [1,2,3]. The Intel team first proposed a separate-absorption-charge-multiplication (SACM) APD structure and achieved a primary responsivity of 0.55 A/W at 1.31 μm and high gain-bandwidth product of 340 GHz, which was equivalent to commercial III-V compound APDs [4]. Nevertheless, applications like sensing, laser radar, and medical diagnostics require APDs with higher responsivity and wider detection bands [5,6]. Limited by the energy band structure of Ge, its absorption coefficient starts to drop sharply from 1.55 μm, resulting in the low responsivity of traditional Ge/Si APDs in the C+L band. To enhance light absorption, surface-illuminated APDs usually adopt thick absorption layers. However, this also leads to an increased carrier transit time and reduced 3-dB bandwidth. The vertical surface-illuminated SACM-APD proposed by the IME achieved a primary responsivity of 0.3 A/W and a 3 dB bandwidth of 13 GHz at 1.55 μm with a 1 μm thick Ge layer [7]. Therefore, how to realize high-speed and high-responsivity surface-illuminated APD in the C+L band is an urgent issue to be addressed.

A diverse range of measures, including on-silicon lead-free halide perovskite single-crystalline thin film (SCTF), nanostalagmite structures, Ge nanowires, grating structures, graphene interlayer, reflector mirror, and tensile strain, have been investigated to enable high-performance photodetectors [8,9,10,11,12,13,14]. Of them, tensile strain is considered an effective measure to enhance the optical properties of Ge [15]. Several methods have been proposed for introducing the strain in Ge, such as thermally induced strain during epitaxial growth on Si, the use of a stressor layer, etc. [16]. Among these, the application of silicon nitride as a stressor layer has been widely studied, mainly because of its compatibility with CMOS processes and flexible control of stress transfer to attain high tensile strain in Ge [17]. Moustafa El Kurdi et al. used an omnidirectional SiN stressor to coat Ge microdisk, achieved a biaxial tensile strain of 1.75%, and transformed Ge into a direct bandgap semiconductor [18]. Besides, Lin Y et al. employed a recessed SiN stressor to elevate the tensile strain of Ge to 0.56% in germanium-on-insulator (GOI) metal-semiconductor-metal photodiodes, with an extra 10~60% rise in the absorption coefficient of C+L band [19].

In this paper, a high-performance surface-illuminated SACM Ge/Si APD was designed. The SiN stressor layer was used to introduce tensile strain into Ge to strengthen its absorption coefficient in the C+L band. The influence of the SiN stressor on the in-plane strain *ε*_xx_ distribution in Ge was explored and uniformly distributed *ε*_xx_ of 0.59% amplitude was obtained. Furthermore, the device structure was optimized considering the constraint relationship between the responsivity and bandwidth. The optimal structure of this design improved the primary responsivity of 0.80 A/W at 1.55 μm, 0.72 A/W at 1.625 μm with a high speed of 17.5 GHz 3-dB bandwidth.

## 2. Design of Si-Based Strained Ge APD

Under the theory of deformation potential [20], the introduction of tensile strain will cause the reduction of *Γ* and L valleys in the conduction band and the separation of light- and heavy-hole valence bands of Ge, attaining lower direct and indirect bandgaps of Ge which can be described as quadratic polynomials of the strain [21]:

(1) $$E_{g}^{LH}=E_{g_{0}}+\delta E_{hy}+\Delta /2-1/4\delta E_{sh}-1/2\sqrt{\Delta^{2}+\Delta \delta E_{sh}+9/4{\left(\delta E_{sh}\right)}^{2}}\;$$

(2) $$E_{g}^{HH}=E_{g_{0}}+\delta E_{hy}+1/2\delta E_{sh}$$

$E_{g}^{LH}$, $E_{g}^{HH}$ are the bandgaps of each valley to the light hole and heavy hole, $E_{g_{0}}$ is the bandgap of bulk Ge. Where $\delta E_{hy}=a\left(\varepsilon_{⊥}+2\varepsilon_{∥}\right),\;\delta E_{sh}=2b\left(\varepsilon_{⊥}-\varepsilon_{∥}\right)$, $\varepsilon_{∥}$, $\varepsilon_{⊥}$ indicate the in-plane strain and the strain perpendicular to the film, respectively, with the relationship $\varepsilon_{⊥}=-\varepsilon_{∥}/1.33$. a and b are the deformation potential constants of Ge, Δ is the spin-orbit separation energy. The direct bandgap absorption coefficient of strained Ge can be expressed as:

(3) $$\left|\alpha \left(hv\right)\right|=A\left(\sqrt{hv-E_{g}^{\Gamma }\left(lh\right)}+\sqrt{hv-E_{g}^{\Gamma }\left(hh\right)}\right)/hv$$

where A is a constant related to the effective mass of the materials. As the bandgap of strained Ge shrinks, the absorption coefficient in its response band increases as well as the response wavelength extends to the L-band [22].

**Figure 1:**
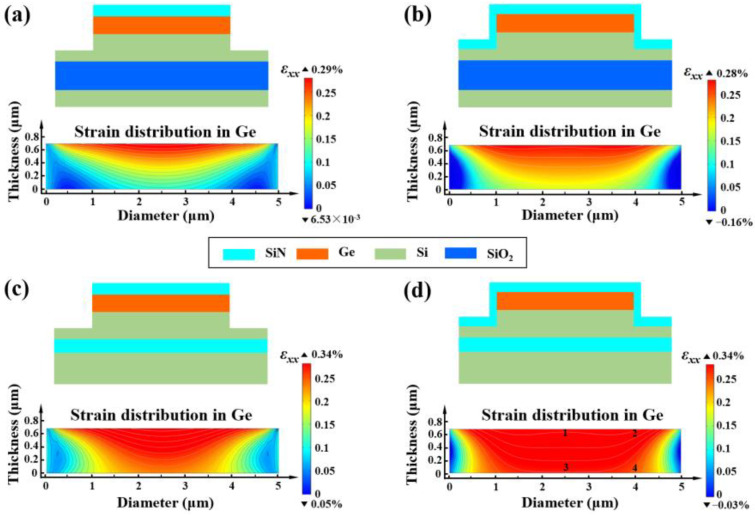
2D cross-sectional view at the centrosymmetric point of the Ge/Si APDs adopting (**a**) structure 1 and (**b**) structure 2 and (**c**) structure 3 and (**d**) structure 4 with strain profiles in Ge.

Since the different thermal expansion coefficients of Ge/Si, the Ge-on-Si epitaxial growth and cooling resulted in an inherent tensile strain of 0.15~0.3% in Ge [23,24], it was assumed that an initial even strain is 0.25% [25]. The location of the SiN stressor will affect the magnitude and uniformity of the introduced strain in Ge. Multi-physics simulation is used to investigate the x-directional strain *ε*_xx_ distribution in Ge transferred from SiN stressor under different device structures. Owing to the centrosymmetric nature of the surface-illuminated APD, *ε*_xx_ can be expressed as an in-plane strain. The diameter of the mesa structure on the N+ electrode is 5 µm, the pre-stress in SiN is set to be 3 GPa. It can be observed that only the upper surface of Ge is subjected to the stress transmitted by SiN stressor in Figure 1a,c. The distribution of *ε*_xx_ in Ge is less uniform. However, because of the addition of SiN stressor on the sides of the mesa and the lower steps, both the left and right Ge are subjected to the stress transfer as well, the evenness of *ε*_xx_ in Ge in Figure 1b,d was significantly enhanced. Furthermore, it can be found that by replacing the SiO_2_ layer in the SOI substrate with SiN stressor, the overall tensile strain in Ge is raised. The maximum value of *ε*_xx_ has been increased from 0.28% to 0.34%. At the same time, Ge is affected by stress transfer from all directions, the strain homogeneity is remarkably improved. Structure 4 has achieved an evenly distributed *ε*_xx_ with an amplitude of 0.34%, which is the most optimal structure. The following will analyze several factors affecting the tensile strain in Ge based on structure 4.

**Figure 2:**
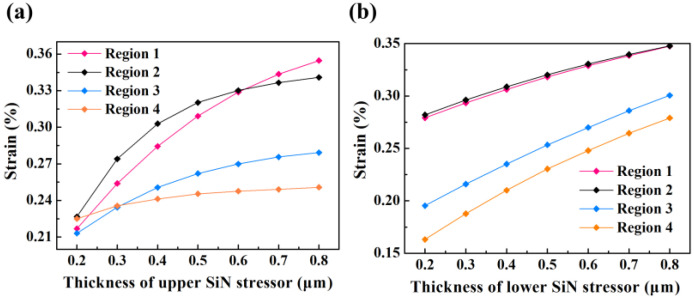
The introduced strain *ε*_xx_ values in regions 1–4 for different thickness of (**a**) upper SiN stressor layer and (**b**) lower SiN stressor layer.

In structure 4, the thickness of the upper and lower SiN stressors will influence the strain distribution in Ge. When the thickness of the upper and lower SiN layers is both 0.6 µm and the sidewall SiN is 0.3 µm, the profile of *ε*_xx_ in Ge is exhibited in Figure 1d. The strain values of *ε*_xx_ in regions 1–4 are 0.329%, 0.331%, 0.270%, and 0.248%, respectively, which revealed a high degree of homogeneity. Moreover, these values increase synchronously with the thickness of the upper SiN stressor (Figure 2a). When the thickness of top SiN layer is only 0.2 µm, the values of *ε*_xx_ in regions 1–4 are 0.217%, 0.227%, 0.213%, and 0.225%, respectively. While as the SiN thickness reaches 0.8 µm, the strain then rises to 0.355%, 0.341%, 0.279%, 0.251%, and the speed of increasing gradually slows down. In addition, the strain at the bottom part of Ge (region 3 and 4) elevates rapidly as the thickness of the underlying SiN layer is added, exhibiting a ~1.75x enhancement (Figure 2b). When the thickness of lower SiN is 0.2 µm, the values of *ε*_xx_ in regions 1–4 are 0.279%, 0.282%, 0.193%, 0.163%, and then increase to 0.347%, 0.348%, 0.301%, 0.279% when the thickness becomes 0.8 µm. The uniformity of the strain distribution is also improved. The thicker the SiN stressor is, the higher the overall strain *ε*_xx_ in Ge. Nevertheless, excessively thick SiN stressor deposition will lead to larger wafer warpage, which is not conducive for process integration. 0.6 µm thickness of the upper and lower SiN layers are selected to obtain up to 0.34% in-plane tensile strain in Ge.

**Figure 3:**
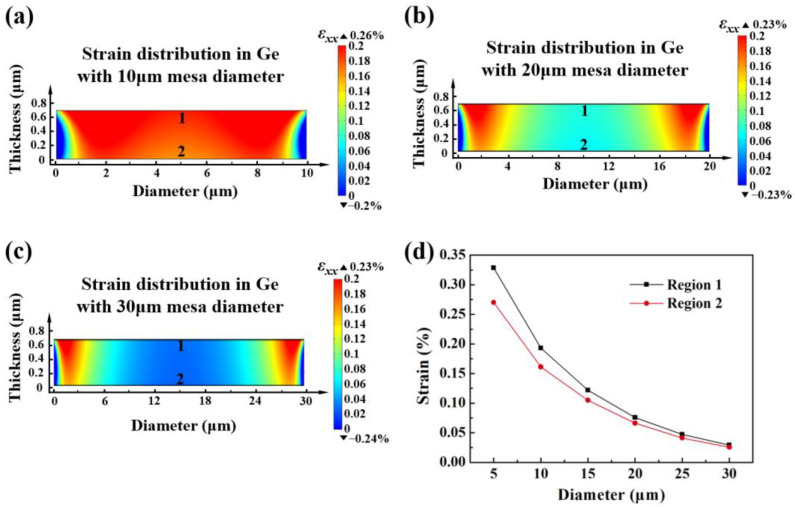
The strain profiles in Ge with (**a**) 10 µm mesa diameter and (**b**) 20 µm mesa diameters and (**c**) 30 µm mesa diameter. (**d**) The value of introduced strain in region 1–2 with various mesa diameters.

Figure 3 simulates the in-plane strain *ε*_xx_ in Ge for structure 4 with different mesa diameters under the same SiN stressor setting. In the previous study, the *ε*_xx_ in Ge was uniformly distributed and reached 0.329% in region 1, the strain in the edge area was slightly higher at 0.34%. It can be seen that when the mesa diameter is 10 µm (Figure 3a), the maximum strain in Ge keeps shifting toward the edge region and the amplitude drops to 0.26%. The strain in the central region of Ge continuously diminishes to the range of 0.05~0.1%, only about 0.23% strain exists in the edge region, upon the diameter of the mesa increases to 20 µm (Figure 3b). Furthermore, when the mesa diameter is 30 µm, the low strain region in the middle of Ge expands and remains the range of 0~0.05%, the edge region still maintains a strain of about 0.23% (Figure 3c). It can also be seen from the curves of the variation of *ε*_xx_ in region 1 and 2 with mesa diameter in Figure 3d, the larger the size of Ge, the more dispersion of the SiN stress transfer and the lower the overall strain, which is detrimental to the optimization of Ge light absorption.

**Figure 4:**
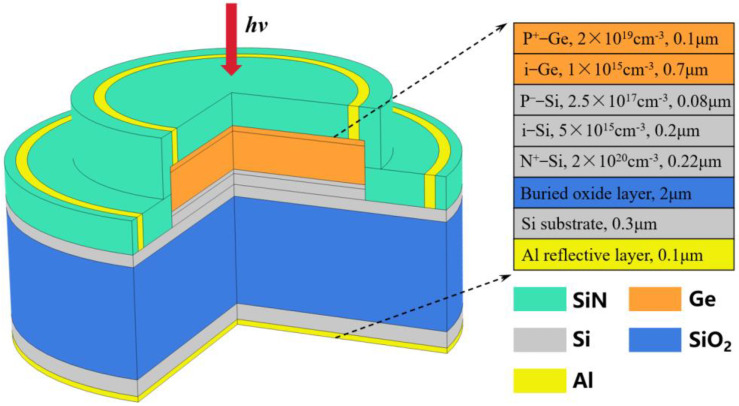
3D schematic of the surface-illuminated SACM Ge/Si APD.

Based on the above analysis, both structures 2 and 4 achieve a relatively uniform strain distribution in Ge. The strain amplitude in Ge of structure 2 can reach 0.53%, while structure 4 has the most homogeneous strain distribution and a maximum magnitude of 0.59%, which is the optimal strain level. Figure 4 presents the three-dimensional structure as well as the doping and thickness of each layer for the SACM APD based on structure 2. Due to the excellent chemical stability of SiN, it can be used as a device surface protection film. SiN also features good optical properties with a refractive index of around 2.0. The SiN on the photosensitive surface can be applied as an anti-reflective coating (ARC) layer to reduce the reflection of incident light utilizing the thin film interference principle. Since aluminum can attain over 85% reflectivity for the light range of 0.4~10 μm [26], a 0.1 μm thick aluminum layer was added to the back of the device as a reflective layer [27]. Under the effect of the resonant cavity formed by the top ARC layer and the bottom reflective layer, the incident light was reflected in it to generate resonance enhancement (RCE) with the goal of raising the responsivity of the device [28].

## 3. Results and Discussion

### 3.1. IV Characteristics

Although structure 4 is better than structure 2 in terms of homogeneity and strain amplitude, achieving the optimal strain level, structure 2 is more manufacturable in practical fabrication. Consequently, the device performance simulations employing technology computer-aided design (TCAD) software will be carried out for the surface-illuminated APDs with structures 2 and 4.

**Figure 5:**
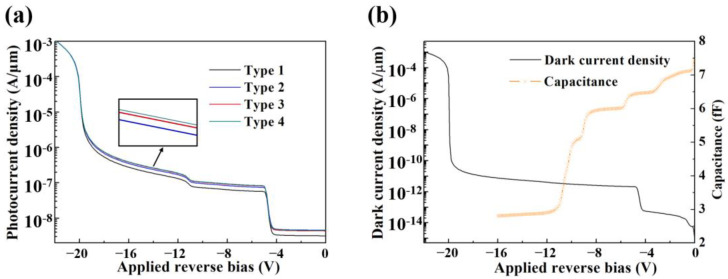
(**a**) Photocurrent density of four types of devices vs. applied reverse bias voltage. (**b**) Dark current density and capacitance vs. applied reverse bias voltage.

Figure 5a shows the curves of the photocurrent density as a function of applied reverse bias voltage for the four types of APDs in Table 1. According to Figure 5b, the dark current reaches 10^−4^ A at −20.2 V, which can be defined as the avalanche multiplication voltage *V*_br_. The photocurrent density is achieved at a normal incidence of 2 W/cm^2^ light intensity at 1.55 μm wavelength. At 0.95*V*_br_, the photocurrent density of type 1 is only 1.07 × 10^−6^ A/μm, the corresponding device optical absorption is 45.1%. When the resonant cavity is added to form RCE, the photocurrent density of type 2 reaches 1.35 × 10^−6^ A/μm, the optical absorption increases significantly to 62.3%. The tensile strain amplitude in Ge of type 3 is up to 0.53% as well as the photocurrent density and absorption rate reach 1.45 × 10^−6^ A/μm and 67.2%, separately. Because type 4 employs the optimal strain simulation structure, the strain in Ge is as high as 0.59% with 1.47 × 10^−6^ A/μm photocurrent density and 68.2% light absorption. The introduction of the RCE structure together with the SiN stressor notably enhances the light absorption of the device. The gap between type 3 and type 4 in terms of photocurrent and device optical absorption is inconspicuous.

Analyzing the rectification behavior of the device for dark current density variation displayed in Figure 5b. When the bias voltage *V*_bias_ is above −5 V, the depletion region exists only in Si at a relatively low dark current. As the *V*_bias_ increases, the depletion region expands into Ge, leading to a rise in dark current due to the generation-recombination current in the absorption region [29]. The punch-through of Ge occurs between −12 and −5 V, and the capacitance characteristics of APD also confirm that Ge is completely depleted at −12 V with the minimum value of 2.82fF.

### 3.2. Responsivity Characteristics

**Figure 6:**
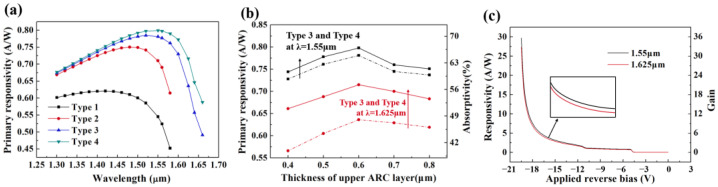
The primary responsivity of (**a**) four device types at various wavelengths and (**b**) type 3 and type 4 with different thicknesses of ARC layer at 1.55 and 1.625 μm wavelength. (**c**) DC photoresponsivity and gain vs. applied reverse bias voltage characteristics of type 4 at 1.55 and 1.625 μm wavelength.

The primary responsivity (gain = 1) *R*_p_ can be obtained by responsivity testing of p-i-i-n device without a charge layer. The *R*_p_ of the four types of APDs in the incident light wavelength range of 1.3~1.66 μm is illustrated in Figure 6a. For type 1, considering only the initial strain in Ge, its *R*_p_ at 1.55 μm is only 0.54 A/W. Type 2 demonstrates a high *R*_p_ of 0.71 A/W at 1.55 μm after the addition of the RCE. By contrast, type 3 extends the response band to L band upon the adoption of SiN stressor, where the *R*_p_ rises to 0.78 A/W at 1.55 μm and 0.64 A/W at 1.625 μm. As the improved strain uniformity and amplitude in Ge of type 4, the *R*_p_ is overall higher than that of type 3, especially in the L-band, reaching 0.80 A/W at 1.55 μm and 0.72 A/W at 1.625 μm.

The SiN served as the top mirror in the RCE structure is not as thick as possible. As shown in Figure 6b, it can be found that when the thickness of upper SiN is 0.6 μm, the *R*_p_ of type 3 and type 4 are maximized at 1.55 and 1.625 μm, and the highest light absorption rate of type 3, 4 reach 67.3%, 68.3% at 1.55 μm and 54.7%, 59.7% at 1.625 μm, respectively. Hence, the optimal thickness of SiN as the upper surface ARC layer is 0.6 μm, which is consistent with the size used in the strain simulation.

The responsivity and gain at 1.55 and 1.625 μm wavelength incident light of 2 W/cm^2^ light intensity as a function of *V*_bias_ is further investigated for type 4 device (Figure 6c). At 0.95*V*_br_, the responsivity along with the gain at 1.55 μm is 14.7 A/W and 18.4, while those at 1.625 μm are 13.5 A/W and 18.8.

### 3.3. Trade-Off between Responsivity and Bandwidth

Since the photon propagation path of the surface-illuminated APD is in the same direction as the carrier multiplication path, there is a restrictive relationship between the responsivity and bandwidth [30]. Then three groups of different Ge absorption layer thicknesses (0.6 μm, 0.7 μm, 0.8 μm) were chosen for type 3 and type 4 to simulate the *R*_p_ and 3-dB bandwidth *f*_3-dB_, select the appropriate thickness to attain high-performance device.

**Figure 7:**
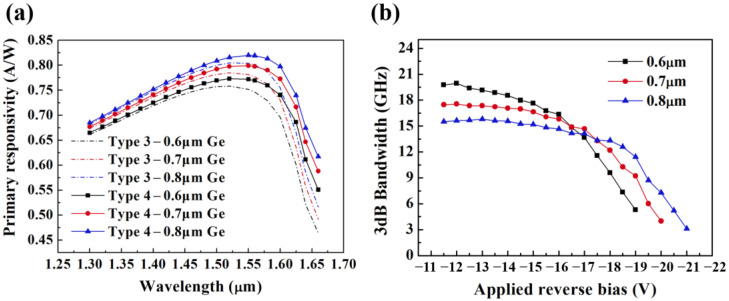
(**a**) The primary responsivity and (**b**) 3-dB bandwidth of APDs based on type 3 and type 4 with different Ge absorption layer thicknesses (0.6, 0.7 μm, 0.8 μm).

Figure 7a presents the changing curves of *R*_p_ in the 1.3~1.7 μm band for APDs with different absorption layer thicknesses. As the Ge thickness grows, the length of the absorption region grows and the *R*_p_ goes up. The *R*_p_ of type 3 and type 4 at 1.55 μm increases from 0.75, 0.77 A/W to 0.80, 0.82 A/W, and the *R*_p_ at 1.625 μm rises from 0.60, 0.69 A/W to 0.67, 0.74 A/W along with the thickness goes up from 0.6 to 0.8 μm. Figure 7b illustrates the variation of the *f*_3-dB_ values as a function of *V*_bias_ for different Ge thickness. Since type 3 and type 4 utilize the same SACM structure, the *f*_3-dB_ values are almost identical. As the thickness of Ge absorption layer increases from 0.6 um to 0.8 um, the carrier transit time rises with the *f*_3-dB_ at low gain and decreases from 19.8 to 15.5 GHz continuously. *R*_p_ and *f*_3-dB_ values of the surface-illuminated APD vary in opposite trends with absorption layer thickness. Hence, a Ge absorption layer with 0.7 μm thickness is chosen as a compromise to realize high-responsivity and high-speed Ge/Si APD.

Table 2 summarizes the reported experimental performance parameters of Ge/Si APDs. As the waveguide-integrated APDs can elongate the length of the absorption region to capture high responsivity without affecting the response speed. The waveguide APD using thin silicon achieved 25 GHz *f*_3-dB_ and 0.75 A/W at 1.55 μm [31]. However, the operating voltage gain value inversely proportional to the thickness of the multiplication zone is 12, lower than the conventional surface incidence APDs. Moreover, this structure is not as capable of collecting scattered light signals in the domain of freely spatial communication as the surface incidence structure [32]. The surface-illuminated APD designed from this work exhibits a high bandwidth simulation value of 17.5 GHz through reduction of the multiplication region, which is better than the reported surface incident APDs and comparable to the waveguide types. The gain value at 0.95*V*_br_ thus drops to 18 with a gain-bandwidth product (GBP) of 162 GHz. Besides, the *R*_p_ of this design achieves 0.8 A/W at 1.55 μm, which is superior to the reported results in Table 2 and second only to the 0.9 A/W of the APD with the optimized RCE structure by SiFotonics, whose responsivity plummeted in the L band [28]. In stark contrast, the present APD attains an *R*_p_ of 0.72 A/W at 1.625 μm. In summary, there are advantages of Ge/Si surface-illuminated APD with SiN stressor in both responsivity and bandwidth according to the simulation results. Moreover, the effect of RCE structure on the responsivity is also very considerable, and the design of the resonant cavity can be carefully optimized subsequently to further enhance the device’s performance.

## 4. Conclusions

A high-performance surface-illuminated Ge/Si APD applied to C+L band light detection is designed using SiN stressor to improve the optical properties of Ge. Two structures with a homogeneous distribution of tensile strain in Ge and magnitude up to 0.53% and 0.59%, respectively, are proposed. The adoption of the resonant cavity substantially boots the light absorption in the response band of APD, and the elevation of tensile strain in Ge slightly raises the responsivity of the device in the C-band, simultaneously extending the detection band to the L-band. For the optimized design of Ge/Si APD with 0.59% amplitude tensile strain in Ge captures an *R*_p_ of 0.80 A/W at 1.55 μm and 0.72 A/W at 1.625 μm, the *f*_3-dB_ at a low gain is maintained at 17.5 GHz. This work demonstrates that mechanical strain engineering is a promising scheme to achieve high-speed, high-responsivity Ge/Si APDs in the C+L band.

## Figures and Tables

**Table 1:** Four types of Ge/Si APDs with various structures.

Type	Device Structure	RCE	SiN Stressor
1	Structure 2	×	×
2	Structure 2	√	×
3	Structure 2	√	√
4	Structure 4	√	√

**Table 2:** Performance comparison of reported Ge/Si APDs.

Ref.	λ (μm)	Device Type	Device Structure	*V*_br_ (V)	*I*_d_ (μA)	*R*_p_ (A/W)	Gain	*f*_3-dB_ (GHz)	GBP (GHz)
[31]	1.55	Waveguide-integrated	Vertical SACM	−10	1	0.75	12	25	276
[33]	1.55	Waveguide-integrated	Lateral SACM	−12	100	0.78	36	27	300
[34]	1.55	Waveguide-integrated	Lateral PIN	−11	100	0.49	13	16	210
[35]	1.55	Waveguide-integrated	Three-Terminal	−6	1000	0.48	15	18.9	/
[7]	1.55	Surface-illuminated	Vertical SACM	−29.0	5	0.3	39	13 *	310
[28]	1.55	Surface-illuminated	Vertical SACM	−28.5	3	0.9	12	7 (M = 12)	84 *
[36]	1.55	Surface-illuminated	Vertical SACM	−27	100	0.26	34.5	/	/
This work	1.55	Surface-illuminated	Vertical SACM	−20.2	7.85	0.80	18	17.5	162
	1.625					0.72			

* These values were not clearly reported but were inferred from the reports.

## Data Availability

Not applicable.
